# Structural Stability of the SUPER304H Steel Used in Energetics

**DOI:** 10.3390/ma15020455

**Published:** 2022-01-07

**Authors:** Lucie Pilsová, Jakub Horváth, Vladimír Mára

**Affiliations:** Department of Materials Engineering, CTU in Prague, Karlovo náměstí 13, 120 00 Prague, Czech Republic; jakub.horvath@fs.cvut.cz (J.H.); vladimir.mara@fs.cvut.cz (V.M.)

**Keywords:** austenitic stainless steel, SUPER304H, reheaters, superheaters, sigma phase, precipitation, carbides

## Abstract

This paper describes the influence of technological treatments (i.e., bending or welding) on the structural stability of SUPER304H austenitic steel used in reheaters and superheaters in fossil fuel power plants. Although the worldwide trend is transitioning to green power sources, the lifetime of existing power plants has to be prolonged until the transition is complete. Experimental material was tested in as-received state (straight tubes), bends, and homogeneous weld joints. Part of the specimens was solution-annealed after the technological operation. Afterwards, all the samples were thermally aged in furnace (650, 675 and 700 °C) for 7560–20,000 h. For comparison, bent specimens were placed at experimental sites on an operating powerplant for 10,000+ h. The long-term aging causes the formation of Cr-based carbides on the grain boundaries along with the Fe-Cr sigma phase. Combination of elevated temperature and residual stress accelerates formation of the sigma phase. This can be prevented by solution-annealing after bending. Mechanical properties were evaluated by Vickers hardness and tensile tests. The microstructure was observed using light optical microscopy (LOM) and scanning electron microscopy (SEM) with the energy-dispersive X-ray detector (EDXS). Electron backscatter diffraction (EBSD) and X-ray powder diffraction (XRPD) were used to characterize the brittle phases.

## 1. Introduction

The operating conditions in the most exposed parts of the ultra-supercritical boilers are reaching the limits of the steel’s capabilities as a working part, even when speaking of high alloyed heat-resistant steels. It also applies to the SUPER304H steel, which belongs to the complex-alloyed austenitic steels. This steel was chosen as a material for the renovation of reheaters and superheaters in fossil fuel power plants and also for the construction of new ones. The manufacturer delivers this material only in the form of reheater tubes, which are manufactured and heat treated in a way to achieve the best properties given by the standard. Each subsequent process (cold working, welding, etc.) leading to disruption of the as-received state results in deterioration of material properties [1]. 

The arrangement of the superheater requires a lot of cold bending associated with large deformations [1]. This leads to a change in the mechanical properties; however, it is not clear what impact will have the previous deformation on the long-term operation of boiler. In the case of welding, as reported in [2], if the correct filler material is not chosen, it can cause the formation of delta ferrite. It can be a suitable nucleation site for undesirable brittle phases after long term operation [3]. 

The first changes in the microstructure are beginning after 5000–10,000 h of operation, and their significance depends on the operating temperature. All published articles agree on the formation of Cr_23_C_6_ carbides at the grain boundaries, while in the base material there are present primary strengthening particles (NbC and NbN) that remain stable. Depending on the operational parameters (i.e., time and temperature), Z-phase NbCrN and tetragonal sigma phase particles can be also found [4]. The sigma phase itself may appear in various systems such as: Fe-Cr, Fe-Mo, Fe-V, Fe-Mn, Fe-Cr-Ni, Fe-Cr-Mo, Fe-Cr-Mn and Fe-Cr-Ni-Mo. As was mentioned above, the M_23_C_6_ carbides are present almost from the beginning of the operation time. The sigma phase presence can be significant after longer periods of time (up to 70,000 h) at temperatures ranging between 620 and 700 °C [5]. Earlier formation of the sigma phase may be initiated by residual stress in the material, such as shot peening of the surface [6] or the creep test conditions [7]. 

From the historical point of view, the predecessor of S304H steel, the AISI 304H steel, contains the delta ferrite areas which are suitable for the sigma phase nucleation (difference in the surface energy, Cr-rich regions) and at suitable conditions can be fully replaced by the sigma phase. Since SUPER304H steel is fully austenitic, the formation of the sigma phase is slower and less pronounced. The articles [6,7] suggest that the acceleration of the sigma phase growth in SUPER304H steel is caused by a certain combination of the temperature, time and residual stress. This is also base for the studies in the presented article. 

Since the 1997, SUPER304H steel has been used as a superheater tubing system material in Japanese power plants [8]. The same trend was then followed in the new facilities in China [9] and Poland [7]. In the Czech Republic, it has been discussed since 2016 for the purpose of modernizing the former power plant blocks. Therefore, the heterogenous weld joints with older materials, such as martensitic P92 steel [10] and ferritic T91 steel [11], are often analyzed.

## 2. Materials 

The experimental material SUPER304H was delivered in the form of seamless reheater tubes (cold finished) with outer diameter OD = 38 mm and wall thickness WT = 6.3 mm (Sumitomo Metal Industries, Ltd.; Amagasaki, Japan). The welding filler material, Thermanit 304H Cu (Böhler Welding, Düsseldorf, Germany), was in the form of coiled wire with diameter d = 0.8 mm, according to the manufacturer’s specifications, it is suitable for joining austenitic creep resistant CrNi(N)-based steel grades with high temperature corrosion resistance. Both materials meet the nominal composition (see Table 1) given by the standard VdTÜV 550.

## 3. Methods

### 3.1. Grinding and Polishing

Samples were cut with a MSX255 metallographic saw (LECO Corp., St. Joseph, MO, USA) and mounted in the Struers EPOFIX epoxy resin. The samples intended for electron microscopy were mounted in Technotherm3000 conductive powder using the MX400 automatic mounting press (LECO Corp., St. Joseph, MO, USA). The samples for light optical microscopy (LOM) were ground on water-cooled SiC paper ending at grit 1000 and polished with a 0.05 μm Al_2_O_3_ suspension Buehler Master Prep. Samples for scanning electron microscopy (SEM) and EBSD analysis were after standard grinding and polishing electrolytically polished using an automatic DC power source LectroPol (Struers ApS, Ballerup, Denmark) with the setup: 25 V, 2 s, flow rate 20 L/s.

### 3.2. Etching

Various etching techniques were used to reveal the microstructure. The basic procedure was electrolytic etching in a 10% aqueous solution of oxalic acid. Depending on the state of the material, the conditions ranged from 1.5 V (thermally aged material) to 25 V (as-received or annealed). This process can be altered with chemical etching using various combinations, such as Glyceregia or Acetic Glyceregia (15 mL of of HCl, 10 mL acetic acid, 5 mL HNO_3_ with 2 drops of glycerol).

Highly deformed material or thermally aged is better etched with the nitric acid electrolyte (40 mL of H_2_O and 60 mL of HNO_3_). The power source was always set at 1.5 V, because a lower voltage causes cathode reduction. This process does not always reveal twins inside the grains, as oxalic acid or chemical etchants do. 

For revealing the brittle phases containing chromium (coarsened carbides, sigma phases) is suitable sodium hydroxide electrolyte. This technique needs a fine adjustment of the etching parameters because the phases can easily get overetched (needless to say, this is true for all methods). Special care needs to be taken when rinsing the sample. Suitable is washing with methanol and drying using an absorbent pad (compact cotton wool, thick layer of tissues,...). Particles are then highlighted, and when observing using LOM, they are colored blue, green, red or white. This method is suitable for electron microscopy and EDS analysis only when the brittle phases are preserved.

The sigma phase was also revealed using Beraha II tint etchant (48 g of NH_4_HF_2_, 400 mL of of HCl, 800 mL distilled water) mixed just before use with 0.5 g of K_2_S_2_O_5_ per 100 mL. Before etching, the samples were electropolished using a perchloric acid-based electrolyte (50 mL of HClO_4_, 15 mL of of HNO_3_, and 1000 mL methyl alcohol). The sample was immersed for 30 to 100 s until the reddish surface appeared. 

For basic electropolishing and etching, the Struers A2 electrolyte containing perchloric acid was used. Automated electrolytic polishing was performed with the setup: 25 V DC, 2 s, flow rate 20 l/s. When etching thermally aged samples with this electrolyte, the sigma phase can be observed as white colored with dark highlighted edges.

### 3.3. Microscopy and Related Analyses

LOM images were taken using microscopes Neophot32 (Karl Zeiss, Jena, Germany), Epiphot (Nikon, Tokyo, Japan) and DSX1000 (Olympus Corp., Tokyo, Japan). SEM was performed using the Jeol JSM-7600F scanning electron microscope (JEOL, Tokyo, Japan) equipped with detectors for EDS (Oxford X-Max 50 mm^2^, Oxford Instruments, Abington, UK) and EBSD (HKL Nordlys, Oxford Instruments, Abington, UK). Quantitative EDS point analysis on the Beraha etched samples was performed with a Tescan Lyra3 scanning electron microscope (Tescan, Brno, Czech Republic). The results of the EBSD analysis in the IPF maps and the grain size map were processed using the ATEX software (Version 3.x, Metz, France) [14]. The Bruker D8 Discover powder X-ray diffractometer equipped with Co anode and Lynx Eye detector was used for phase identification and quantification. (Bruker, Billerica, MA, USA)

### 3.4. Bending

For the purpose of this paper, two types of bends with radii R60 and R80 mm were made using the hydraulic NC tube bender Prefekt WE (Schwarze-Robitec GmbH, Cologne, Germany). Samples were extracted from the top of the bend, where the highest deformation was presumed. The simulation of the maximum deformation in the bends was calculated using the finite element method software PMD and GFEM processor (Vamet s.r.o., Prague, Czech Republic).

### 3.5. Welding

The set of homogeneous weld joints of the pair of tubes was made using the TIG orbital arc welding method with Ar shield gas and Thermanit 304HCu filler material. The average parameters are presented in Table 2. Additional testing for delta ferrite presence was done using the Ferritgehaltsmesser 1.054 ferrite meter (Institut Dr. Förster, Reutlingen, West Germany).

### 3.6. Mechanical Properties

HV10 hardness measurement was done using an automated hardness tester Duramin 40 AC3 equipped with a Vickers indenter (Struers ApS, Ballerup, Denmark). The color hardness maps were plotted using the data analysis and graphing software OriginPro (Version 2021b SR1, Northampton, MA, USA). Tensile testing at room and elevated temperature was carried out with the Instron 5800R Tensile and Compression Test System with constant crosshead speed v = 0.5 mm/s (Instron, Norwood, MA, USA).

### 3.7. Heat Treatment

The as-received tubes were according to the material sheet [12] “heat treated at 1150 °C” after rolling. Part of the samples in form of bends and weld joints were solution annealed at 1130 °C for 15 min with cooling in water. In this case, the samples are referred to as heat-treated (HT) or annealed.

### 3.8. Laboratory Aging

To gain a closer understanding of the microstructural changes in the material, the samples were long-term aged in a resistance furnace at 650, 675, 700 °C for 7560, 15,000 and 20,000 h, respectively, in the air atmosphere. The temperature was continuously checked with a type K thermocouple placed in the center of the furnace chamber.

### 3.9. Power Plant Material Exposure

**Figure 1:**
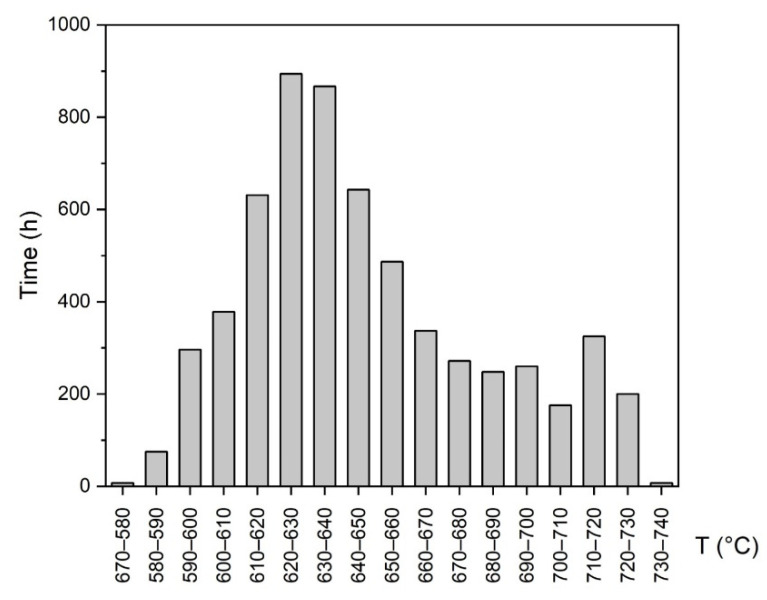
Power plant boiler operation temperature histogram.

For comparison with laboratory aged samples, a group of bends was placed in the K3 boiler in the Dětmarovice Power Plant, Czech Republic. These samples were exposed to the flue gas flow at a wide temperature range (see Figure 1) for 10 484 h.

## 4. Results

The results are divided into sections according to the technological procedure or testing parameter. When needed, for better context, the results are combined in one section.

### 4.1. Bending

**Figure 2:**
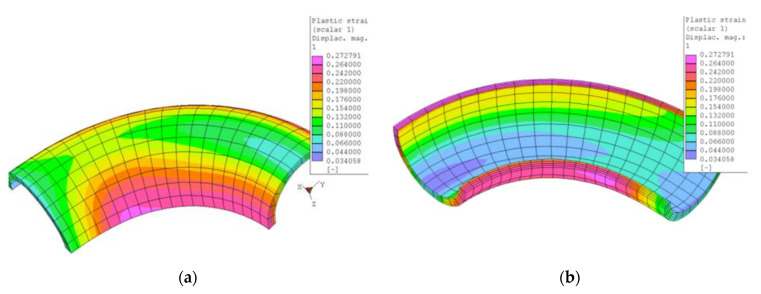
Isoplanes of plastic deformation after bending (**a**) outer and (**b**) inner views in the FEM PMD software.

Except for the neutral axis of the bend, the size of the bending radius defines the amount of plastic deformation. In the case of the sharper radius R60, the maximum deformation was calculated about 27%. In Figure 2 there is a simulation of the bend with maximum deformation approximately equal in the areas of the tension-stressed and compression-stressed fibers.

### 4.2. Welding

**Figure 3:**
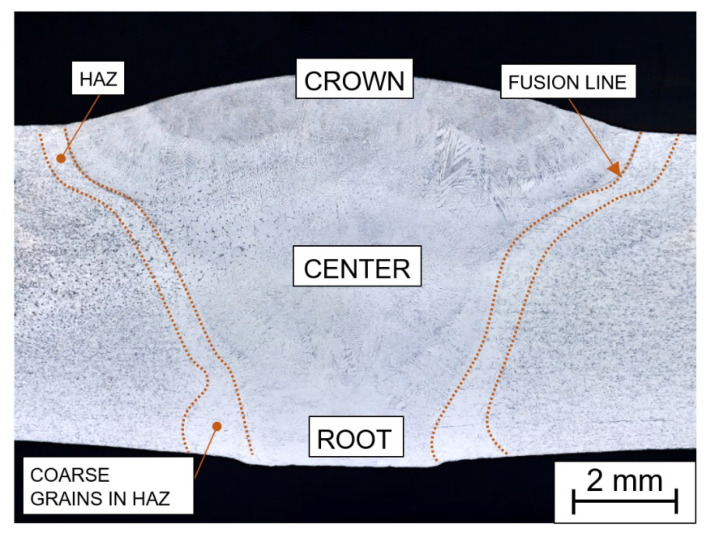
Macrograph of the homogeneous weld, without heat treatment, immersion etched in Glyceregia.

The homogeneous butt weld (Figure 3) consists of the weld metal (WM), heat-affected zone (HAZ) with clearly visible fusion line (FL). The HAZ is divided into the coarse-grained part (most pronounced in the root area) and the fine-grained zone that continuously leads to the base material SUPER304H.

### 4.3. Tensile Test

**Figure 4:**
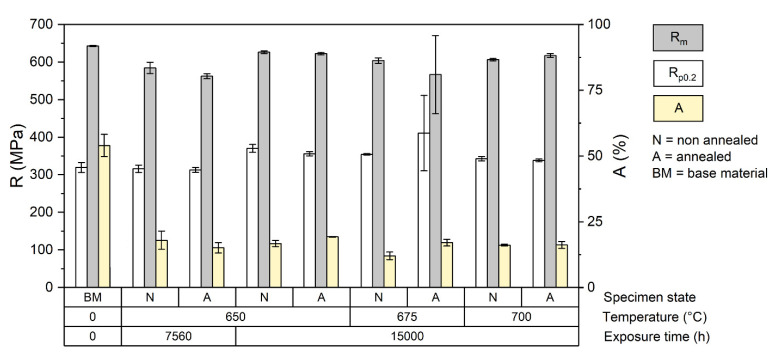
Results of the tensile test at RT of the weld joint specimens.

The tensile test of the base material was performed at room temperatures of (RT) and at elevated temperature 650, 675 and 700 °C. The results of the RT tensile test are shown in Figure 4.

For the filler material used for welding, the minimum tensile strength is 590 MPa [13], which is in accordance with the standard for the SUPER304H [12]. The homogeneous weld joints reached tensile strengths in the range of 623–645 MPa. From this point of view, all the weld joints had met the standard. All specimens failed in the heat affected zone. 

Thermally aged weld specimens with heat treatment had higher tensile strength (622 ± 3 MPa) after 15,000 h at 650 °C than after 7560 h at 650 °C (562 ± 6 MPa). This is probably caused by precipitation of carbides in the microstructure and variance of the grain size. The average elongation decreased to the 16% compared to base material with A = 55% and the weld joint after heat treatment with 34%.

Thermally aged weld specimens without heat treatment showed slightly different behavior. With absence of recovery and recrystallization, the tensile strength is higher, but with loss of ductility. Specimens just after welding had an average tensile strength of 20 MPa higher than those with heat treatment. The changes in an elongation remained in similar manner (A = 18%) as heat treated, but the difference is more pronounced after 15,000 h at 650 ° C where the average tensile strength reached 604 ± 5 MPa but with a very low elongation of 12%. Thermal aging may partially imitate the effect of heat treatment, but the temperature was still very low for appropriate homogenization and restoration of the former properties. 

### 4.4. Hardness

#### 4.4.1. Bends

The hardness maps are presented as polar graphs with marked segments in degrees around the cross sections of the tubes. Due to the deformation after bending, the final shape is no longer circular.

**Figure 5:**
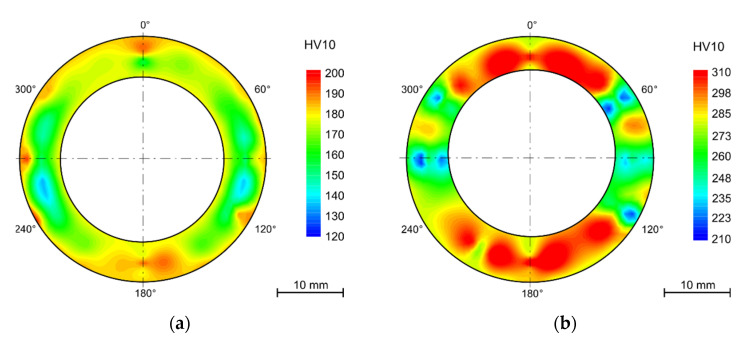
(**a**) As-received (solution annealing 2 min); (**b**) as-received after bending, without HT.

In the as-received state, there is quite variance in the hardness values from the outer diameter to the inner diameter of the tube (Figure 5a). This is probably due to the manufacturing process and the very short annealing time (2 min). The difference between inner and outer hardness is about 25 HV10. This trend was preserved after bending among those samples that were not annealed after bending (Figure 5b).

**Figure 6:**
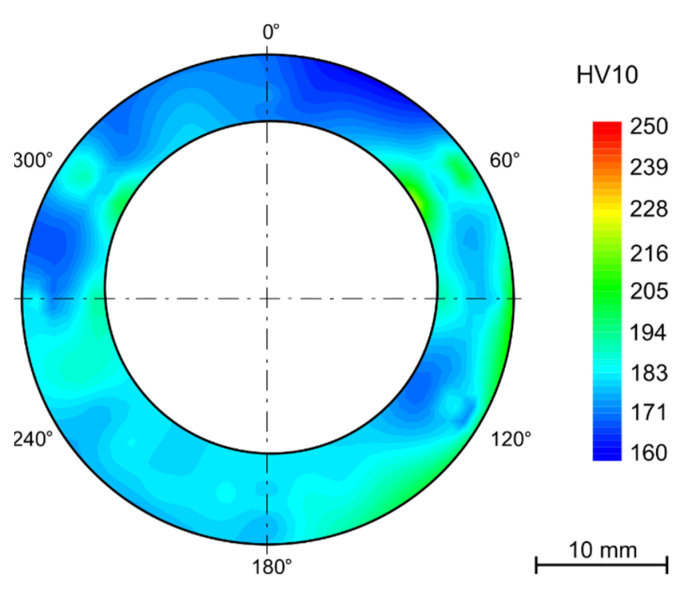
Solution annealed after bending (1130 °C/15 min/water).

The solution annealing after bending lead to the hardness increase of 80–100 HV10 in tension- and compression-stressed fibers and a decrease of approximately 50 HV10 in the neutral axis (Figure 6). This process eliminated the influence of the bending process and restored the as-received structure with average hardness of 180 HV10. The variation of hardness between the outer and inner diameters was also eliminated.

**Figure 7:**
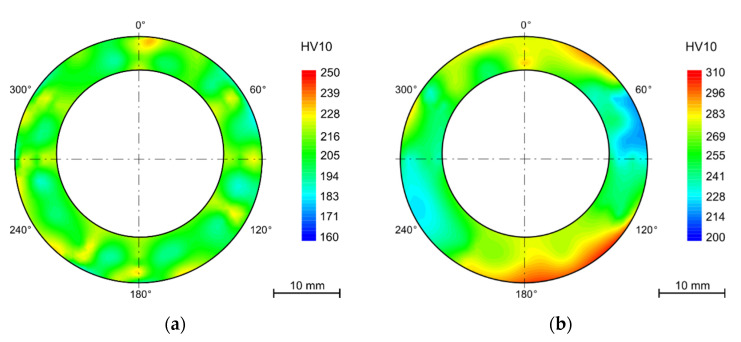
Heat exposure in powerplant 10,484 h (**a**) solution annealing after bending (1130 °C/15 min/water); (**b**) without HT.

After bending and in some cases after annealing, the samples were thermally aged for 7500 h in the laboratory furnace at 650 °C. Among the annealed samples, this even led to a decrease in hardness to 202 ± 3 HV10. In the case of samples that were thermally aged but were not annealed after bending, the average hardness decreased from 299 ± 22 HV10 to 265 ± 18HV10. With the absence of the annealing process, the difference in the hardness values across the tube wall remained. The same pattern can be observed in the hardness maps of the samples exposed to the operating conditions (Figure 7).

The hardness gradient throughout the wall thickness and the areas with the highest hardness in the most deformed parts (tension-stressed and compression-stressed) also remains after thermal aging after 15,000 h. Nevertheless, the decrease of hardness is not linear and with a longer time of aging is not so significant.

#### 4.4.2. Welds

Because the temperature of 700 °C is the limit value and is not recommended by the manufacturer, the homogeneous welds were tested at 675 °C for 7560 h.

**Figure 8:**
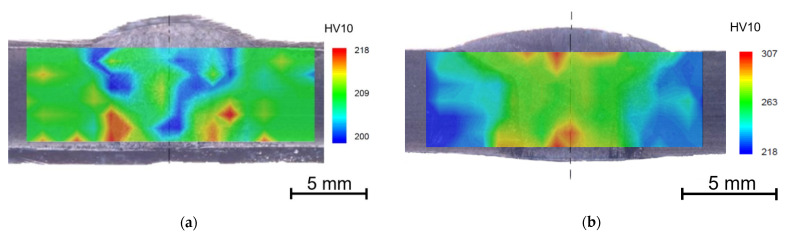
Hardness maps of the annealed weld joint after aging (**a**) 650 °C/7560 h, (**b**) 675 °C/7560 h.

Figure 8 shows the HV10 hardness maps along the entire weld joints with solution annealing (1130 °C/ 15 min/water) and then aged for 7560 h at 650 and 675 °C.

Although the weld metal is almost identical to the base material and the entire weld joint was heat treated, the aging temperature of 675 °C led to a higher increase in hardness in the weld metal and also in the heat affected zone than in the base material. It can lead to the conclusion that every intervention in the as-received state leads to the deterioration of the initial properties.

### 4.5. Grain Size

**Figure 9:**
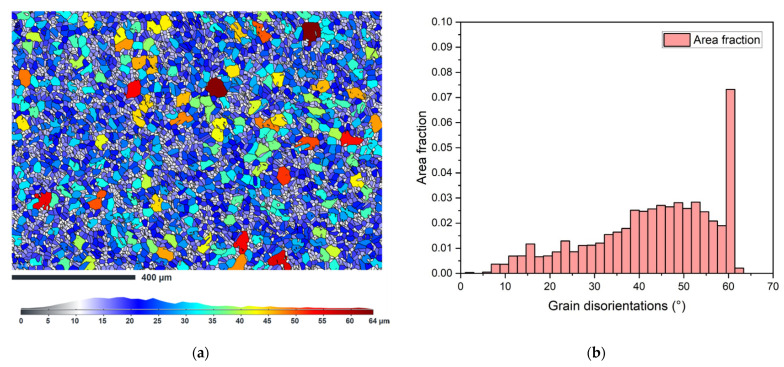
(**a**) Grain size map of the sample as-received, longitudinal section; (**b**) total area fraction of the disorientation of the grain boundaries.

The grain size was determined from the EBSD analysis of the as-received sample. The legend in Figure 9a shows that most of the grains are in size number G 8–9 (average diameter 0.0221–0.0156 mm). Locally coarse grains had a size of up to G 5 (grain diameter equal to 0.0625 mm). The histogram in Figure 9b shows the total area fraction of grain boundaries disorientation in the analyzed area, where high angle GBs are dominant along with twins corresponding to ~ 60 °.

The grain size in the bent samples determined by the ASTM E112-13 intercept method is shown in Table 3.

The grain size in the weld joints (A—solution-annealed, N—nonannealed) was evaluated using the ASTM E112-13 comparison procedure. The row arrangement in the Table 4, Table 5, Table 6 and Table 7 follows the weld joint design (see Figure 3), considering the left and right heat-affected zone separated from the weld metal by the fusion line FL. The grain sizes in brackets refer to the areas close to the surface where the grain size differs significantly.

### 4.6. Microstructure

**Figure 10:**
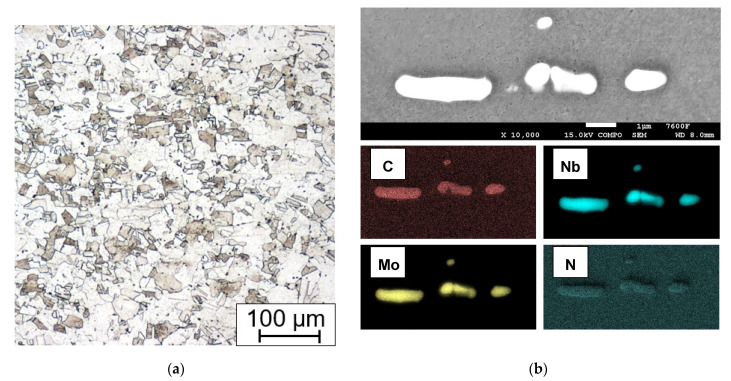
As-received state, (**a**) electrolytic etching (15 V DC, 40 s) in 10% aqueous acetic acid (LOM micrograph); (**b**) strengthening particles type Nb(C,N) EDXS element mapping in the longitudinal section.

Every state of the material after heat treatment and technological operation was documented using LOM and for some specimens SEM to obtain further details. The as-received material (Figure 10) consists of polyedric austenitic grains with dispersed strengthening particles of Nb(C,N). In the cross section of the tubes, the Nb(C,N) particles seem to be evenly dispersed, but in the longitudinal section they form bands due to the manufacturing rolling process. 

**Figure 11:**
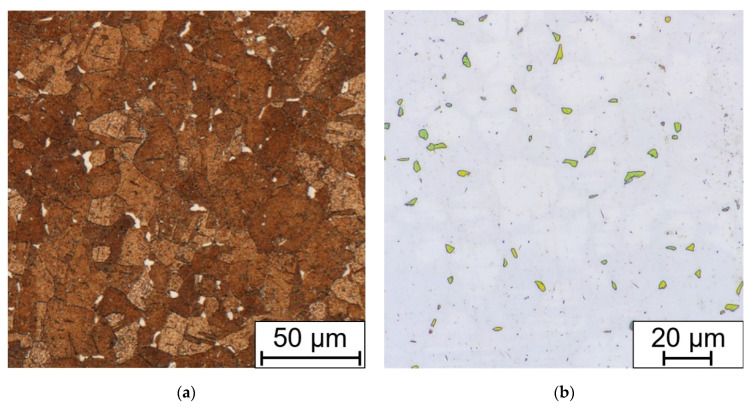
Comparison of (**a**) Beraha II immersion etched and (**b**) electrolytic NaOH etched (1.5 V DC, 1 s) bend sample without HT, after 7560 h at 650 °C.

After the long-term aging (or heat exposure in the real operation service) changes begin on the grain boundaries. Chromium MX and M_23_C_6_ carbides begin to precipitate. After longer periods, about 5000 h and more (depending on the temperature and the state of the material, deformation or previous heat sensitization after welding), the brittle phases appear, in this case the sigma phase (Figure 11).

**Figure 12:**
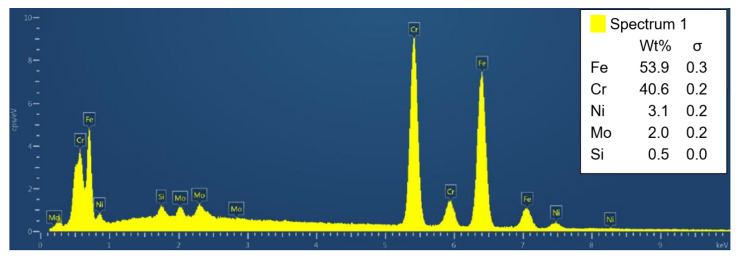
Indexed peaks of the EDXS point analysis of the sigma phase particle.

The presence of sigma phase in the Beraha II-etched sample was confirmed by the EDXS point analysis (Figure 12). Thus, it can be concluded that the sigma phase in SUPER304H steel consists mainly of Fe and Cr followed by smaller amounts of Ni and Mo. All EDXS analyzes result in a similar manner.

**Figure 13:**
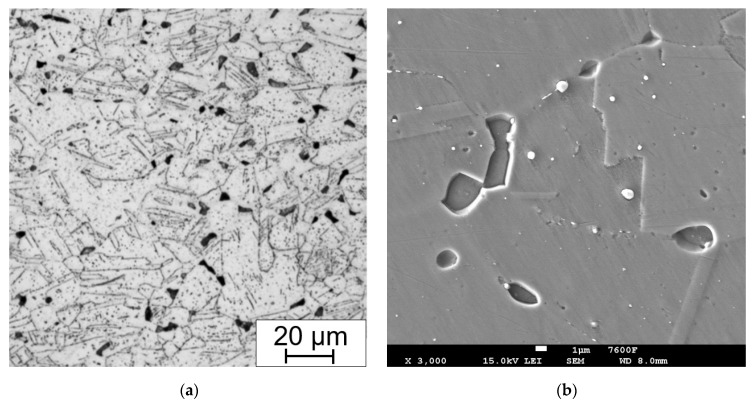
Example of (**a**) LOM and (**b**) SEM micrograph with not sufficient preparation (particles removed with electrolytic etching in 10% aqueous acetic acid, 5 V DC, 10 s) for EDS analysis of the bend sample without HT, after 15,000 h at 650 °C.

For the basic observation, the electrolytic etching in the oxalic acid solution electrolyte was fully sufficient. However, for the SEM observation, the oxalic acid etching completely removed the sigma phase (see Figure 13).

**Figure 14:**
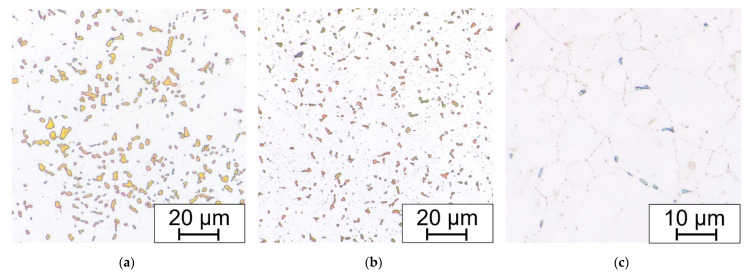
(**a**) Compression-stressed fiber; (**b**) tension-stressed fiber; and (**c**) neutral fiber; bend without HT, all aged 650 °C/15,000 h and electrolytic etched in 20% aqueous NaOH (1.5 V DC, 1 s).

The large influence of the deformation after bending without annealing is shown in Figure 14. Long-term aging at 650 °C for 15,000 h leads to formation of the brittle phases in the microstructure. The amount of sigma phase varies depending on the fiber position in the bending process. The best conditions for the growth of the sigma phase were in the compression-stressed fibers. The least affected area is the area closest to the neutral fiber.

**Figure 15:**
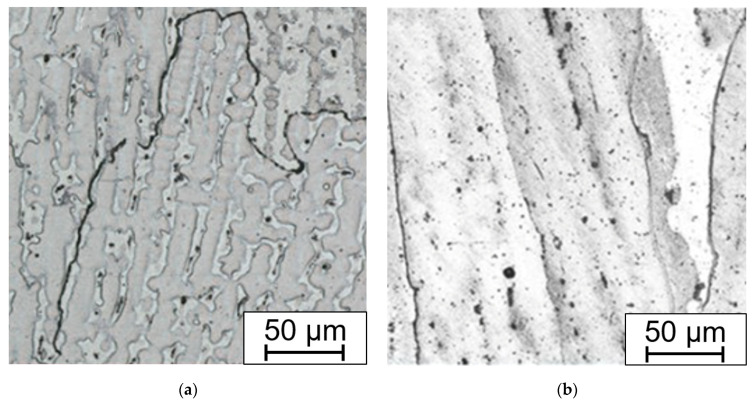
Microstructure of the weld metal Thermanit304Cu (**a**) after welding; (**b**) after solution annealing. Electrolytically etched in 10% aqueous acetic acid (5 V DC, 10 s).

The weld joint structure in both states, with and without solution annealing, consists of gamma austenite. The microstructure of samples without HT consists of grains with ragged areas inside lined with fine precipitates. These areas, mainly after slight overetching, may be misinterpreted as delta ferrite (Figure 15a).

The absence of delta ferrite was confirmed by the Ni and Cr equivalent drawn into the Schaeffler diagram, where the amount of delta ferrite equals zero. The control measurement using a calibrated ferrite meter also showed that delta ferrite is missing.

**Figure 16:**
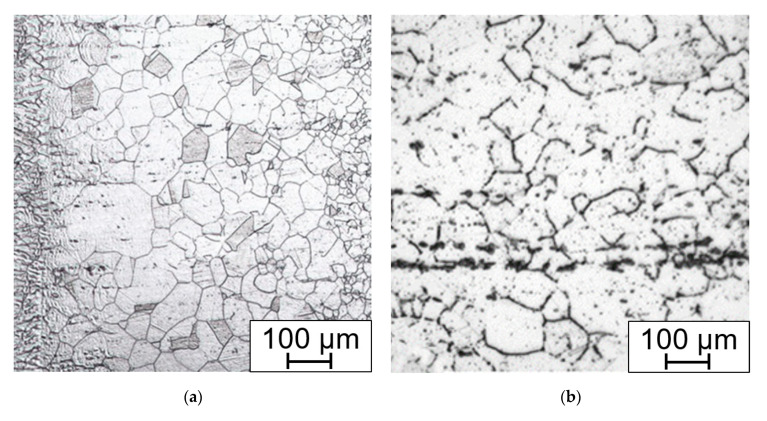
Weld without annealing, (**a**) HAZ with fusion line on the left; (**b**) HAZ, precipitation on the GB, rows of the primary carbides Electrolytically etched in 10% aq. acetic acid (5 V DC, 10 s).

Except for coarse grains close to the fusion line (Figure 16a), there are fine precipitates inside the solid solution and at the grain boundaries (Figure 16b). Very pronounced precipitation is present in the HAZ approx. 2 mm from the fusion line. Carbides and carbo-nitrides are present in the form of discontinuous wrappings around the grain boundaries. Inside the fine grains in the recrystallized zone there are also very fine precipitates. The primary strengthening particles remained unchanged. The precipitation decreases with the growing distance from the HAZ with the gradual transition.

**Figure 17:**
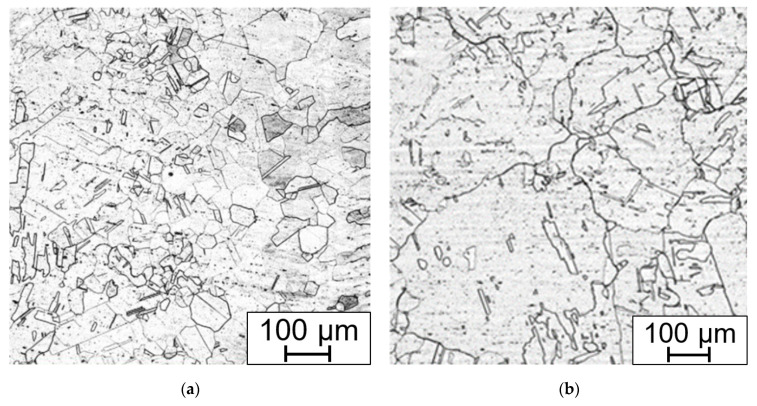
Annealed weld (**a**) HAZ and fusion line on the right; (**b**) annealed areas with coarse grains and annealing induced twining. Electrolytically etched in 10% aq. acetic acid (5 V DC, 10 s).

Annealing leads to the dissolution of the carbidic wrappings on the grain boundaries (Figure 17a), but also creates irregular-shaped areas close to the base material, which can be up to 5 mm wide, contrasting with the fine-grained areas. In these coarse grains (up to G −1 which corresponds to the mean diameter of 0.5 mm), there are various twin boundaries with different size and orientation (Figure 17b).

**Figure 18:**
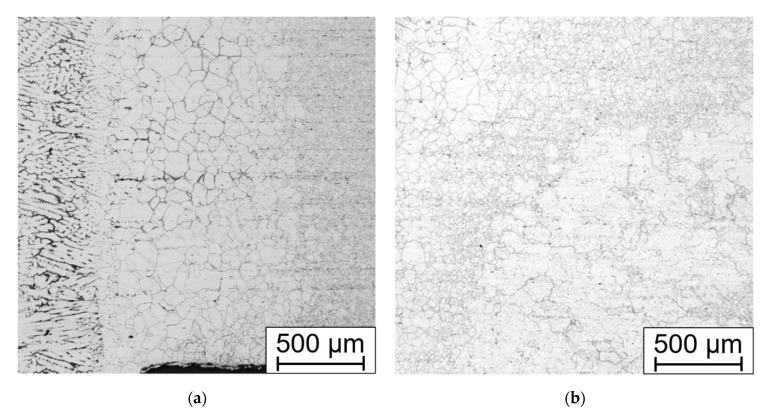
Root of the weld (**a**) without HT; (**b**) after HT with coarse grain areas close to the HAZ on the left. Electrolytically etched in 10% aqueous acetic acid (5 V DC, 10 s).

Therefore, the solution annealing that was suitable for the bends is not suitable for the welds. Heat treatment leads to dissolution of the grain boundary-weakening particles but creates coarse grained areas in the root of the weld. The width of the heat-affected zone among the welds without HT (Figure 18a) is 0.2 to 1.9 mm and after HT it is 0.2 to 1 mm, but with locally coarse grains near the HAZ (Figure 18b). 

After long-term aging at 650 °C there are carbides on the grain boundaries and the sigma phase only locally; it is more pronounced after 700 °C. However, the main influence on fracture toughness deterioration is probably the grain size inhomogeneity. 

### 4.7. XRPD and EBSD

**Figure 19:**
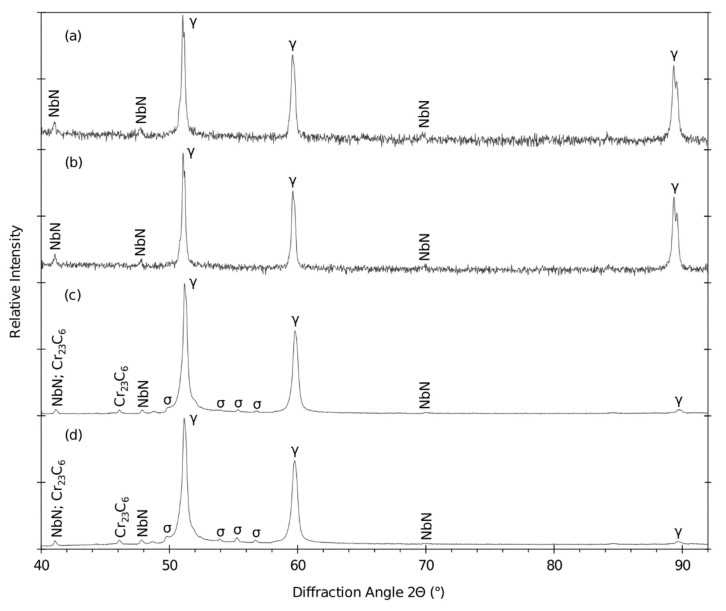
Diffraction patterns of the bends (**a**) in the annealed state (A), (**b**) and not annealed state (N), (**c**) nonannealed after 650 °C/7560 h and (**d**) nonannealed after 650 °C/15,000 h.

XRPD was done on the base material after bending in the annealed and nonannealed state (see diffraction patterns in Figure 19) and on two long-term aged samples extracted from the top of the bends. The two samples were long-term aged at 650 °C for 7560 and 15,000 h.

The diffraction profiles of the base material show significant peaks that represent the austenitic matrix with NbN particles. According to the EDXS measurement, there is also a certain content of C. Due to its overall lower content in the steel (0.03 wt.%) compared to the N content (0.85 wt. %), the N content dominates in the NbN/C particles.

The weight fraction and the lattice parameters of the identified phases in the long-term aged samples are summarized in Table 8.

**Figure 20:**
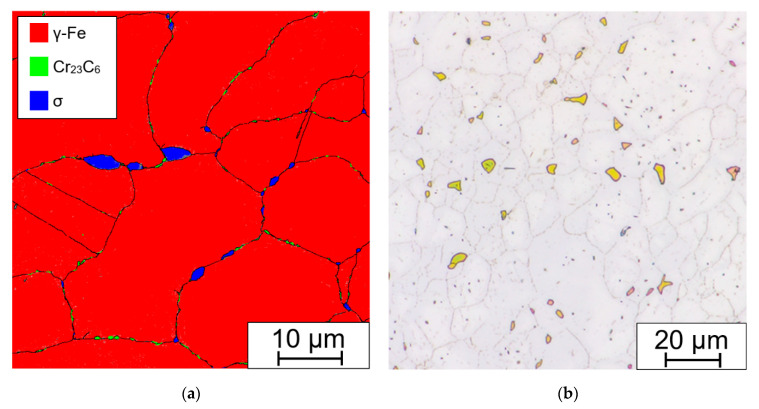
Sample aged at 675 °C/ 20,000 h: (**a**) phase map from EBSD; (**b**) optical micrograph, etched in 20% NaOH electrolyte (1.5 V DC, 1 s).

The presence of Fe-Cr sigma phase and Cr_23_C_6_ carbides in the microstructure was also confirmed by EBSD analysis (see Figure 20a). For comparison, the etched microstructure with highlighted sigma phase is shown in Figure 20b. 

The Fe-Cr sigma phase corresponds to the tetragonal space group P42/mnm (no. 136) and Cr_23_C_6_ carbides to the cubic space group Fm-3m (no. 225). It can be seen that the coarse Fe-Cr sigma phase forms at the grain boundary triple junction and also along the grain boundaries, while finer Cr_23_C_6_ carbides are present solely along the grain and Σ3 {111} twin boundaries.

## 5. Discussion

The hardness measurement showed variation in hardness across the wall of the tubes, although the manufacturer declares solution treatment after rolling. The hardness is higher by the outer diameter than in the middle of the wall thickness and then slightly higher by the inner surface. This state did not change after the R60 or R80 bend. The gradient of the hardness values is preserved after long term aging in the laboratory furnace. This phenomenon may bring certain bias to the nondestructive testing (NDT) methods, in this case surface hardness measurement, used during the regular checks.

The strengthening effect has anticipated proportionality to the amount of cold working deformation, which is similar in size in both tension-stressed and compression-stressed fibers. The amount of deformation is reflected in the increase in hardness of 50–60 HV10. After thermal aging at 650 °C for 10,000 h, the hardness decreased by 40 HV10 while in the neutral fiber, the decrease was only about 15 HV10. This leads to the conclusion that higher deformation has a significant impact on the structural stability of the steel and then annealing after bending is required. 

Isotermic aging compared to operation conditions shows only slight differences in hardness and other observed properties. In both cases the M_23_C_6_ carbides precipitation occurred at all aging times and temperatures. The increase in hardness of the annealed samples after long-term aging is caused by the aforementioned M_23_C_6_ and sigma phase precipitation. In accordance with [7], this negatively affects mechanical properties, in this case evaluated by the static tensile test. The evolution of mechanical properties is directly bound to microstructural changes; these changes during the high temperature exposure are based on precipitation hardening that would be positive if the particles did not coagulate and/or coarse. In article [5] are presented results of the operation-aged tubes at average 616 °C for 54,750 h and 68,550 h respectively, with no sigma phase detected. This is in agreement with our findings about sigma phase above 650 °C. 

The importance of solution annealing after bending was also proved by XRPD analysis with the focus on the brittle sigma phase content. The long-term aged sample (675 °C/7560 h) without bend contained 0.26 wt. % of the sigma phase. This amount is almost on the detection limit of the device. In contrast, the bends without solution annealing contained 1.39 wt. % of the sigma phase after one year at 650 °C. After the time equal approximately to the two-year operation, the amount of sigma phase almost tripled. The acceleration of the growth of the sigma phase is clearly caused by plastic deformation after cold working.

In the coarse-grained HAZ close to the fusion line grains of size up to G 3.5 can be found, whereas in the base material it is G 8. The HAZ of the nonannealed weld contains carbidic wrappings on the grain boundaries. After the solution annealing (1130 °C/15 min/water) these carbides are fully dissolved. On the other hand, in the root area close to the HAZ there were observed randomly placed coarse grain (up to G -1) areas with twinning. In the weld metal, the cellular substructure is dissolved into the solid solution. From a concentration point of view, the solution annealing leads to homogenization, but also brings the grain coarsening. 

Average hardness values do not significantly vary between the annealed and nonannealed welds. In the HAZ of the nonannealed weld, the highest values of hardness are found close to the fusion line with the coarse grains, whereas the annealed welds have the highest values of hardness at the border of HAZ and the base materials in the coarse grain areas. Depending on the used filler material, delta ferrite may form in the weld metal as in the case of welds in [2] and [7] did.

The results of the RT tensile test of the as-welded specimens are in accordance with results R_p0.2_ = 349.6 MPa ± 10.2 and R_m_ = 614.6 ± 11.7 MPa presented in [2]. On the contrary, the rupture in [2] occurred in the base material, while in our case in the HAZ. The tensile test of the specimens with homogeneous welded joint regardless of solution annealing showed that after long-term aging (650 °C/7560 h) the yield strength decreased while the hardness value increased. This suggests certain changes in the toughness of the material. The average elongation has decreased from A = 54% to 17%. This precipitation-caused behavior is in accordance with [7], but the weakest point was the T24 steel in the dissimilar weld joint. 

## 6. Conclusions

The discussed results can be summarized in the following conclusions:

- Hardness varies between the tubes’ wall in the as received state despite heat treatment after rolling. The hardness is higher for the outer and inner surfaces (approx. 200 HV10) and lower in the middle of the wall thickness (160 HV10).
- The acceleration of the sigma phase growth is clearly caused by the plastic deformation after cold working. Solution annealing is then a crucial operation. The suggested parameters 1130 °C/15 with cooling in water were fully sufficient.
- Isotermic aging compared to operation conditions (note that the exposure time in the power plant also includes planned outage periods) shows only slight differences in hardness (approximately 20 HV10 in the middle of the wall thickness) and other observed properties. The short period of exceeding the 700 °C temperature during the service operation had no major impact on the stability of SUPER304H steel.
- The suggested solution annealing parameters of 1130 °C/15 min/water were fully sufficient for the treatment of the bent samples, but in the case of the weld joints, it caused unfavorable grain growth close to the HAZ root area.

## Figures and Tables

**Table 1:** Nominal composition (wt. %) of SUPER304H steel and welding filler material Thermanit 304 H Cu [12,13].

Material	C	Si	Mn	P	S	Cu	Cr	Ni	Nb	N	B	Al
SUPER304H	0.03	<0.3	<1.0	<0.040	<0.010	3.0	18.0	9.0	0.45	0.85	0.005	0.017
Thermanit 304 H Cu	0.1	0.4	3.2	- *	-	3.0	18.0	16.0	0.4	0.2	-	-

* Elements marked “-“ are not specified.

**Table 2:** Welding procedure characteristics.

Layer	Welding Speed (mm/min)	Current (A)	Arc Voltage (V)	Preheat/Interpass (°C)	Arc Energy (J/mm)	Heat Input * (J/mm)
1	33.0	99.6	8.8	14	1597	958
2	28.8	89.3	9.5	128	1767	1060
3	25.8	86.5	9.5	148	1911	1147

* Coefficient of efficiency for the 141 method η = 0.6.

**Table 3:** Mean lineal length L (mm) of the grain intercept represented by the grain size number G.

Mean Lineal Length L (Mm)	After Bending	650 °C/7560 h	650 °C/15,000 h	650 °C/18,500 h
Direction 0°	0.0116	0.0143	0.0082	0.0082
Direction 90°	0.0112	0.0128	0.0087	0. 0087
**Avg number G**	9.5	9.5	9.5	10.5

**Table 4:** Grain size G in the N sample, aged at 650 °C for 7560 h.

Location	BM-Left	HAZ-Left		HAZ-Right		BM-Right
		Further Area	FL	FL	Further Area	
crown	6.5	6	5.5	not visible	6	6
center	6.5	7	2	3	5	6.5
root	6.5	6.5	3	not visible	3 (4)	7

**Table 5:** Grain size G in the A sample, aged at 650 °C for 7560 h.

Location	BM-Left	HAZ-Left		HAZ-Right		BM-Right
		Further Area	FL	FL	Further Area	
crown	7.5 (1.5)	7	6.5	not visible	6.5	7.5
center	6.5	6.5	5	5	6.5	7.5
root	7.5 (6)	1	3	4	not visible (4)	8

**Table 6:** Grain size G in the N sample, aged at 700 °C for 15,000 h.

Location	BM-Left	HAZ-Left		HAZ-Right		BM-Right
		Further Area	FL	FL	Further Area	
crown	8.5	7	5	6	7	8
center	8.5	6.5	5	4.5	6	8.5
root	8.5	7	3.5	3.5	5.5	8

**Table 7:** Grain size G in the A sample, aged at 650 °C for 15,000 h.

Location	BM-Left	HAZ-Left		HAZ-Right		BM-Right
		Further Area	FL	FL	Further Area	
crown	8	6.5	6.5	6.5	5	7 (3.5)
center	7.5	4 *	2	3.5	5	7.5
root	7.5 (6)	4.5 (1)	not visible	4	1	7.5

* Strong inside-grain precipitation.

**Table 8:** Results after the Rietveld refinement.

Sample	Phase	Weight Fraction (%)	Lattice Parameter (Å)
N-650 °C-7560 h	Austenite	93.24	a = 3.604
	NbN	1.13	a = 4.436
	Cr_23_C_6_	0.78	a = 10.619
	Fe-Cr sigma	4.85	a = 8.806 c = 4.581
N-650 °C-15,000 h	Austenite	93.24	a = 3.604
	NbN	1.13	a = 4.439
	Cr_23_C_6_	0.78	a = 10.62
	Fe-Cr sigma	4.85	a = 8.810 c = 4.585

## Data Availability

Not Applicable.
